# Predictive factors affecting axillary lymph node involvement in patients with breast cancer in Duhok: Cross-sectional study

**DOI:** 10.1016/j.amsu.2019.07.011

**Published:** 2019-07-10

**Authors:** Ayad Ahmad Mohammed

**Affiliations:** Department of Surgery, College of Medicine, University of Duhok, Nakhoshkhana Road 8, AM-1014, Kurdistan Region, Duhok, Iraq

**Keywords:** Breast cancer, Axillary lymph nodes, Staging of breast cancer

## Abstract

**Background:**

Breast cancer is the most common type of cancer affecting women during their life, there are many histological types of breast cancer that have different biological behaviors. Tumors have different genetic and molecular differences which affect the expression of various hormone receptors.

**Patients and methods:**

The aim of this study is to show the factors that determine the axillary lymph node involvement in patients with breast cancer in Duhok city.

A total number of 479 female patients with breast cancer of various histological types, immunohistochemical characteristics, and clinical stages were included in this study. These patients underwent modified radical mastectomy and axillary lymph node dissection.

**Results:**

The mean age of our patients was 48.24 years, the mean number of the positive lymph nodes were 2 lymph nodes, the median size of the tumor was 30 mm. A significant correlation was found with the size of the tumor and the estrogen receptor status (P values (0.000 and 0.042) respectively, while there was no significant correlation with other factors such as the age, stage of the tumor, grade of the tumor, tumor necrosis, progesterone and HER-2 receptors status.

**Conclusion:**

Size of the tumor and the estrogen receptor status were the most common factors that determined axillary lymph node involvement in patients with breast cancer in our study.

## Highlights

• Breast cancer is the commonest type of cancer affecting women during their life.
• Early diagnosis is the most important step in the management.
• Axillary lymph node involvement is one of the most important factors that affects the long term survival.

## 1 Introduction

Breast cancer is the most common type of cancer that affects women during their life time worldwide. It is the most common cancer affecting women in Iraq according the Iraqi cancer registry [1,2].

Breast cancer comprises multiple histological entities that are thought to have various biological behaviors. Invasive ductal carcinoma not otherwise specified constitutes the commonest type and is estimated to be around 70%, other types comprise the resting percentages. There are approximately 17 special types of breast cancer that are analyzed, each thought to have a different biological behavior [3].

The risk factors for breast cancer include increasing age, early menarche, late menopause, late age of the mother for the first pregnancy, nulliparity, obesity, long term use of the combined oral contraceptive tablets, the presence of some genetic mutations like BRC1 and BRC 2 mutations which may cause cancer at earlier age [1,4].

The American joint committe on cancer is using a clinical staging system which is the Tumor-Node-Metastasis system (TNM). This staging system correlates the size, the extent of the primary tumor and the number of the involved regional lymph nodes, and the status of distant metastasis [5,6].

Estrogen is thought to be the most important factor that cause the breast tissue to develop and differentiate, for this reason it plays a very important role in the development of breast cancer. Estrogen receptor (ER) is expressed in 50–80% of breast cancer tissues depending on the age and the menstrual status. The presence of the ER on the breast cancer tissue increases its responsiveness to the estrogen [7,8].

Progesterone receptor is an ER regulatory protein and it is presence in breast cancer tissue have been associated with improved prognostic outcome [8].

The proto-oncogene of the HER‐2/neu (C‐erbB‐2) is located on the chromosome number 17q which encodes for the generation of a growth factor receptor, amplification of this gene expression is shown to be associated with the development of breast cancer. Expression of this gene may be associated with some other cancers such as gastrointestinal tumors, pulmonary tumors and genitourinary tumors. Over expression of this receptor is shown to be associated with poor prognostic outcome [9].

The hormone expression in breast cancer tissue is variable according to the age, sex and between various ethnic groups. This expression in women tends to increase with age, African –American women have the lowest expression of the hormone receptors than other ethnic groups and Caucasian men have the highest expression of the hormone receptors in the removed breast tissue samples which was shown in a study which compared the African-American race with the Caucasian race [10].

Now many laboratories use the immunohistochemical examinations to describe the presence of some other receptors such as p53, c-erbB-2, Ki-67, vitamin D receptors, thyroid hormone receptors, hepatocyte growth factor, and androgen receptors [[11], [12], [13], [14]].

The treatment of breast cancer is mainly surgical but this depend largely on the stage and the treatment strategies that are available at various centers. The surgical options of treatment may include breast conservation surgery with sentinel lymph node biopsy or the modified radical mastectomy and axillary lymph nodes dissection. The treatment may include chemotherapy and radiotherapy which may be given as neoadjuvant or as adjuvant therapy. Hormonal therapy may be needed in some patients. Early diagnosis and treatment had been shown to reduce mortality by 30% [15].

### 1.1 Ethical committee registration

Ethical committee approval granted from the Duhok Directorate of General Health, Scientific Research Division at the 12th of May 2019 with reference number **12032019**–**2**, email: scientific.research@duhokhealth.org.

The research is registered according the World Medical Association's Declaration of Helsinki 2013 at the research registry at 29th of April 2019, Research registry UIN: 4842.

## 2 Patients and methods

### 2.1 Study design and sampling

Patients with breast cancer were operated with modified radical mastectomy and axillary lymph nodes dissection, samples sent for histopathological and immunohistochemical analysis. Histopathological examination done to define the type and size of the tumor with the number of the involved axillary lymph nodes. Immunohistochemical analysis of the tumor tissue done for the expression of the hormone receptors such as ER, PR, and HER.

479 female patients with breast cancer were included in the study. The subjects were recruited from a single center in Duhok-Iraq in 2018.

### 2.2 Inclusion and exclusion criteria

Female patients who were diagnosed with breast cancer were included in the study. Male patients with breast cancer and patients in whom the data were not complete were excluded from the study.

### 2.3 Statistical analysis

The descriptive purposes of this study is to display any correlation between the number of the positive axillary lymph nodes and various tumor and patient characteristics.

The patients and the tumor characteristics displayed in terms of frequency, mean, median and standard deviations. The correlation done using the 2 tailed *t*-test.

The statistical calculations were done in Statistical Package for Social Sciences (SPSS 25:00 IBM: USA).

The work of this article has been reported in line with the STROCSS criteria [16].

## 3 Results

**Fig. 1:**
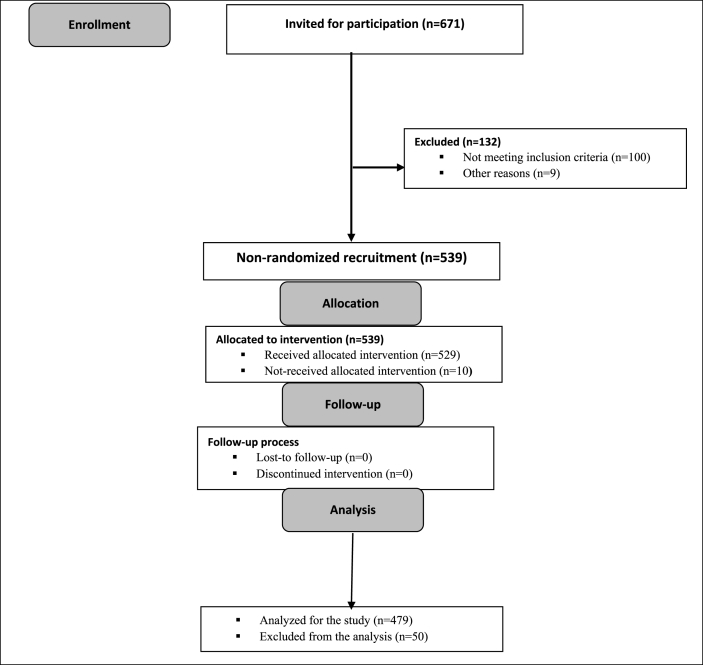
Flow chart of patients' recruitment in the study.

In this study 479 patients met the criteria. Patient enrollment in the study is shown in Fig. 1.

The age range of the patients was between 17 and 80 years with a mean age of 48.24 years. The median size of the primary tumor was 30 mm and the mean number of the involved axillary lymph nodes by the tumor were 2 lymph nodes.

The most common histological type was found to be invasive ductal carcinoma. Bilateral breasts involvement was found in 6 patients (1.3%).

Most patients presented with stage IIIA, IIA, and IIB (25.7%, 24%, and 23.2%) respectively according to the TNM staging system. Detailed patient information and data about the tumor characteristics are shown in Table 1.

**Table 1:** Patients characteristics.

Patients' Characteristics	Frequency Distribution	
Age; M(SD); Range: 17–80 years	48.24	12.44
Size of the tumor (mm); Median	30.00	
Lymph node number; M(SD)	2.0	6.0
**Histological type**; F (%)		
IBC	3	.6
IDC	420	87.7
IDC + PD	3	.6
ILC	39	8.1
MC	11	2.3
PD	2	.4
TC	1	.2
Site of breast cancer; F (%)		
Bilateral	6	1.3
Left	243	50.7
Right	230	48.0
**Stage of breast cancer; F (%)**		
IA	54	11.3
IIA	115	24.0
IIB	111	23.2
IIIA	123	25.7
IIIB	10	2.1
IIIC	56	11.7
IV	10	2.1

Abbreviations: M: mean, SD: standard deviation, F: frequency, IBC: inflammatory breast cancer, IDC: invasive ductal carcinoma, PD: Paget's disease, ILC: invasive lobular carcinoma, MC: mucinous carcinoma, TC: tubular carcinoma.

After correlating the number of the involved lymph nodes with other histological and the immunohistochemical analysis we found a strong correlation with the size of the tumor and the ER status. Other factors have not been shown to have a strong correlation with the axillar lymph node status, Table 2.

**Table 2:** Predictors of axillary lymph nodes involvement in breast cancer.

	Standardized Coefficients	t	Sig.
	Beta		
**TYPE**	.013	.275	.783
**AGE**	-.072	−1.626	.105
**SIZE**	.282	6.325	**.000**
**GRADE**	.094	1.830	.068
**NECROSIS**	.007	.158	.874
**SITE**	-.042	-.941	.347
**ER**	.155	2.044	**.042**
**PR**	-.083	−1.112	.267
**HER**	.027	.550	.583

ER: estrogen receptors, PR: progesterone receptors, and HER2: human epidermal growth factor receptor 2.

The bold number show the predictors.

## 4 Discussion

Axillary lymph involvement had been shown by many authors to be the most important factor that affects the management and the prognosis of breast cancer patients [17].

In our study we included patients that underwent modified radical mastectomy and axillary lymph node dissection. Some centers adopt the axillary sentinel lymph node biopsy in the management of the axilla in breast cancer patients, this may decrease the rate of the axillary lymph node dissection in node negative patients [18].

Ultrasound examination of the axilla is very sensitive but not so specific in detecting axillary lymph nodes involvement, but ultrasound guided biopsy of the suspicious lymph nodes increases the diagnostic accuracy to a higher level that may reach around 80% [19,20].

Magnetic resonance imaging may help not only in differentiating benign from malignant tumors but also it may differentiate aggressive tumors by noninvasive tools [21].

Neoadjuvant chemotherapy is used in the treatment of locally advanced breast cancer and to manage advanced axillary involvement. It can completely treat the axilla from the metastatic disease and the micro-metastasis can be found in only 10% of patients receiving the neoadjuvant chemotherapy [22].

We should differentiate the prognostic factors from the predictive factors. A prognostic factor is defined as any tumor related factor that is detected after surgery and related to the overall survival without the use of any adjuvant treatment, while a predictive factor is defined as any tumor related factor that predicts the response to a specific treatment. Some factors can be used as a prognostic and predictive factors like hormone receptors [23].

All of the mentioned factors may be predictors and prognostic factors which have a very important role in the surgical and the treatment plan, the genetic profile of the cancer cells has been shown to have a critical role in the biological behavior of the tumor [24].

Improved screening will detect the disease at an earlier stage which is the most important prognostic factor that affects survival according to many studies done. Axillary lymph node involvement and the number of the affected lymph nodes is among the very important factors that may predict the survival and also determine the types of the surgical procedure and the chemotherapy treatment plan. In this study we detect a significant correlation with the size of the tumor, while other factors showed no significant relation such as the age and the grade of the tumor [23].

The median tumor size of our patients was 30 mm and the mean number of the involved axillary lymph nodes were 2 lymph nodes. The tumor size had a significant correlation with the total number of the involved axillary lymph nodes (P value 0.000). Studies shown a significant relation with the axillary lymph nodes and the tumor size as what have been shown in our study, other factors that may predict this correlation is the presence of lymphovascular and perineural invasions, these factors together if present have been shown to increase the risk of metastatic disease also [[25], [26], [27]].

The estrogen receptor status was another factor which showed a significant correlation with the axillary lymph node involvement (P value 0.42) this correlation is of positive value in the patient's management. Hormonal therapy improves survival in such groups of patients [28].

In the future scientists may correlate factors other than the tumor size and the number of the involved lymph nodes. They may correlate these factors together with the grade, the hormone receptor and other receptors status, and the molecular differences to adopt a staging system that may guide the treatment strategies [24].

As the number of the involved lymph nodes has a strong correlation with the size of the tumor, so screening programs if done routinely for all the women may detect the cancer at earlier and smaller size and will have a positive impact on the prognosis and the survival rates. This is usually done with the breast self-examination and the mammography which is done for women more than 40 years. The screening program in most parts of the world is targeted for women between 40 and 75 years. Earlier screening is done for women at risk such as with positive family history or genetic predisposition [29,30].

## Ethical approval

Ethical committee approval granted from the Duhok Directorate of General Health, Scientific Research Division at the 12th of May 2019 with reference number 12032019-2, email: scientific.research@duhokhealth.org.

## Sources of funding

No source of funding other than the author.

## Author contribution

Study design: Dr Ayad Ahmad Mohammed.

Data collection: Dr Ayad Ahmad Mohammed.

Data analysis: Dr Ayad Ahmad Mohammed.

Writing: Dr Ayad Ahmad Mohammed.

Final approval of the manuscript: Dr Ayad Ahmad Mohammed.

## Conflicts of interest

No conflicts of interest present.

## Trial registry number

N/A.

## Guarantor

Dr Ayad Ahmad Mohammed.

## Research registration unique identifying number (UIN)

Researchregistry 4842.

## Recommendations

I recommend to have regular screening for women in Duhok city to detect cancer at early stage and patients with estrogen positive tumors should receive hormonal therapy.

## Provenance and peer review

Not commissioned, internally peer reviewed.
